# Assessment of bone health in pre-dialysis CKD patients based on TBS reference range for the Chinese population

**DOI:** 10.3389/fmed.2025.1556782

**Published:** 2025-06-19

**Authors:** Biao Wu, Huanhua Wu, Zheng Wang, Zhiqiang Tan, Bin Guo, Jingjie Shang, Yong Cheng, Chunyuan Zeng, Yuanfang Jiang, Qijun Cai, Jian Gong, Hao Xu

**Affiliations:** ^1^Department of Nuclear Medicine, The First Affiliated Hospital of Jinan University, Guangzhou, Guangdong, China; ^2^Central Laboratory, The Affiliated Shunde Hospital of Jinan University, Foshan, Guangdong, China

**Keywords:** chronic kidney disease, dual energy X-ray absorptiometry, bone mineral density, trabecular bone score, fracture

## Abstract

**Background:**

Pre-dialysis chronic kidney disease (CKD) Patients show a markedly elevated risk of fractures, and BMD assessments offer only limited insights into their bone health. The trabecular bone score (TBS), a newly introduced parameter for evaluating bone microarchitecture, is recommended for studying bone health under this context.

**Methods:**

A total of 46 subjects were included in the control group, and 136 patients were included in the CKD group. All participants underwent laboratory examinations, Dual energy X-ray absorptiometry (DXA) scans, and medical history reviews. The relationships between TBS and demographic characteristics, history of fractures, LS-BMD, FN-BMD, and laboratory parameters were analyzed.

**Results:**

Age, gender, and BMI were matched between the control and CKD groups (P > 0.05). The control group had an average age of 64.96 ± 7.76 years with 27 females (58.70%), while the CKD group had an average age of 64.42 ± 10.90 years with 66 females (48.53%). Among the CKD group, 43 patients had fractures. In both control and CKD participants, when BMD was normal or osteopenia, TBS frequently indicated partially reduced bone microarchitecture or reduced bone microarchitecture, a statistically significant finding (*P* < 0.05). In CKD fracture patients with normal or osteopenic BMD, several patients had TBS classified as degraded or degraded trabecular bone, and among this population, the number of individuals classified as having partially reduced bone microarchitecture or reduced bone microarchitecture based on the TBS (China reference range) is higher than the number classified under the TBS (META reference range). Furthermore, except for the FN-BMD (Osteoporosis) group, TBS-incorporated models significantly improved fracture discrimination across other groups (*P* < 0.05).

**Conclusion:**

In pre-dialysis CKD patients with normal or reduced BMD, TBS is significantly associated with fracture risk. Additional evaluation of bone microstructure using TBS enhances fracture risk identification, particularly in patients with relatively high BMD.

## Introduction

Chronic kidney disease (CKD) is a major global health concern, affecting approximately 11–13% of the population worldwide. Its consequences extend beyond the progressive decline in renal function, also impacting bone health (1). Chronic kidney disease-mineral and bone disorder (CKD-MBD) is a common complication of CKD, which increases the risk of osteoporosis and fractures. The pathophysiology of CKD-MBD is complex, primarily involving metabolic disturbances in vitamin D, phosphate, and calcium (2). Key features of CKD-MBD include reduced bone mineral density and alterations in bone microarchitecture, leading to bone fragility and a higher risk of fractures (3).

Bone biopsy is considered the gold standard for diagnosing CKD-MBD in patients with CKD. However, its clinical application is limited by concerns regarding invasiveness, high cost, and patient acceptance. Dual-energy X-ray absorptiometry (DXA) is widely recognized as the standard method for assessing bone mineral density (BMD) and received a 2B recommendation in the 2017 KDIGO update on CKD-MBD clinical practice guidelines (4). However, the anteroposterior DXA projection for the lumbar spine does not account for factors such as osteophytes, small joint sclerosis, and abdominal aortic calcification (AAC), which may lead to an overestimation of lumbar spine BMD values. This limitation can also result in discrepancies between lumbar spine BMD (LS-BMD) and femoral neck BMD (FN-BMD) measurements. Furthermore, BMD measurements do not capture microstructural changes in bone quality (5, 6).

Trabecular Bone Score (TBS) is a technique used to analyze the structure of standard lumbar DXA images, providing insights into bone microarchitecture that bone mineral density (BMD) alone cannot offer. A higher TBS value is indicative of better bone microstructure, whereas a lower TBS value correlates with weaker bone microarchitecture. Moreover, TBS offers several advantages over non-DXA tests and bone biomarkers (7), including cost-effectiveness, non-invasiveness, and ease of clinical implementation. According to a recent meta-analysis, TBS values are categorized as normal (TBS ≥ 1.35), partially reduced bone microarchitecture (TBS 1.21–1.34), reduced bone microarchitecture (TBS ≤ 1.20) (8). Considering the influence of factors such as ethnicity and geographic region, our team has developed a TBS reference range specific to mainland China. In Chinese men, TBS values are categorized as normal (TBS ≥ 1.39), partially reduced bone microarchitecture (TBS 1.31–1.39), reduced bone microarchitecture (TBS ≤ 1.31). In Chinese women, TBS values are categorized as normal (TBS ≥ 1.35), partially reduced bone microarchitecture (TBS 1.27–1.35), reduced bone microarchitecture (TBS ≤ 1.27) (9).

To date, studies evaluating the use of TBS in patients with CKD remain limited. Existing research indicates that TBS is significantly lower in the dialysis-dependent CKD population, with lower TBS values associated with a higher incidence of fractures or an increased risk of new fractures in CKD patients (10, 11). However, there is a paucity of studies focusing on pre-dialysis CKD patients, particularly regarding the reduction in TBS in those with normal bone mineral density, a topic that remains underexplored. Furthermore, no studies have been conducted on the establishment of a TBS reference range for the Chinese population in relation to bone quality in CKD patients.

Thus, we conducted a retrospective study using DXA to measure parameters such as LS-BMD, FN-BMD, and TBS (L1–4) in pre-dialysis CKD patients. The study involved stratifying CKD patients to evaluate their bone health and explore the clinical significance of these indicators, enabling healthcare providers to identify CKD patients at higher risk for bone health complications in a timely manner.

## Materials and methods

### Study population

**FIGURE 1:**
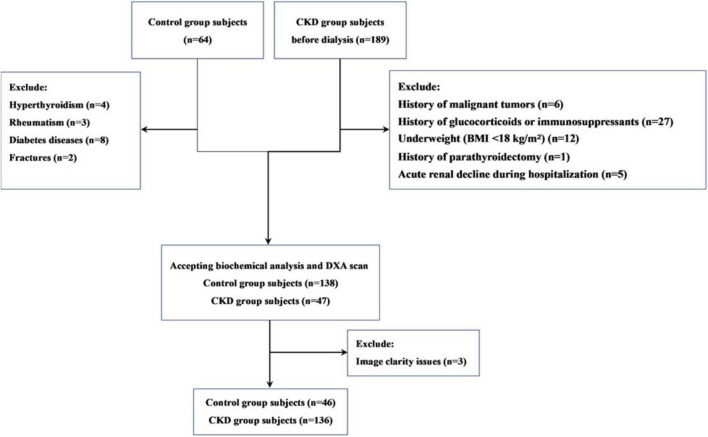
Subject flow diagram.

This is a single-center retrospective observational study. The study participants were pre-dialysis CKD patients recruited from the Affiliated Hospital of Jinan University from December 2021 to December 2022. During this time, we also recruited a control group of individuals with normal kidney function (estimated glomerular filtration rate [eGFR] ≥ 90 mL/min/1.73 m^2^), who were matched with CKD patients based on age and gender. The inclusion criteria for participants were as follows: (1) male patients aged over 50 years or postmenopausal female patients; (2) Patients with CKD who are not yet on dialysis. According to the 2012 KDIGO clinical practice guidelines on chronic kidney disease evaluation and management, CKD is defined as persistent abnormalities in kidney structure or function for over 3 months, with adverse effects on health; (3) signed informed consent. Exclusion criteria included: (1) a history of malignant tumors; (2) use of glucocorticoids or immunosuppressants for more than 3 months in the past 5 years; (3) a history of parathyroidectomy; (4) underweight (BMI < 18 kg/m^2^) or obesity (BMI > 30 kg/m^2^); (5) patients diagnosed with acute renal decline during hospitalization; (6) control group members diagnosed with diseases affecting bone health, such as thyroid dysfunction or rheumatic diseases. In total, 136 CKD patients and 46 controls were included in the study (Figure 1).

### Clinical and anthropometric parameters and biochemical analysis

Demographic and clinical data were collected from prior medical records, including age, sex, smoking history, alcohol consumption history, and medical history (such as diabetes, fractures, and rheumatoid arthritis). Fractures were identified based on clinical history and confirmed by imaging and radiological reports for all patients. Only fractures resulting from minimal trauma within the past 10 years were included in the analysis, while fractures of the fingers, toes, face, and skull were excluded. Biochemical parameters related to bone metabolism were assessed within 48 hours before the BMD and TBS measurements. These included complete blood count, liver and kidney function tests, calcium (Ca), phosphorus (P), serum alkaline phosphatase (ALP), plasma 25-(OH)-vitamin D, and intact parathyroid hormone (iPTH). Throughout the study, the lowest serum creatinine value was used to calculate the estimated glomerular filtration rate (eGFR) using the Chronic Kidney Disease Epidemiology Collaboration (CKD-EPI) formula (12).

### DXA scanning

DXA scans were conducted using the iDXA device (GE Lunar Corp., Madison, WI, United States), adhering to the standard imaging and positioning protocols. Results were presented as grams of bone mineral per square centimeter (g/cm^2^). Prior to routine scanning, quality control procedures were carried out following the manufacturer’s guidelines. In our laboratory, accuracy was recorded in an assessment involving 30 volunteers, and the obtained root mean square coefficient of variation was less than 0.8%. The World Health Organization (WHO) T-score was used to classify bone status for postmenopausal women and men over 50 as normal (T ≥–1.0), osteopenia (–2.5 < T < –1.0), and osteoporosis (T ≤ –2.5). At enrollment, lumbar spine BMD and femoral neck BMD were measured in all CKD patients and controls. The study was categorized by Overall BMD T score, LS-BMD T score, and FN-BMD T score.

### TBS

DXA was used to measure BMD (L1–4) in all participants. All measurements were conducted by experienced operators using the same equipment and standardized procedures, with the operators blinded to the clinical parameters and results. The TBS analysis was performed using TBS iNsight^®^ software (version 3.0.2.0, Med-Imaps, Bordeaux, France) as a grayscale texture index. It involved analyzing the spatial organization of pixel intensities in the lumbar spine (L1–4), which reflects the difference in X-ray absorption intensity between the damaged bone trabeculae structure and the normal bone trabeculae configuration (7). In this study, we categorized bone microstructure based on the TBS (L1–4) ranges derived from our previous research in the Chinese population: for males, normal (> 1.39), partially reduced bone microarchitecture (1.31–1.39), and reduced bone microarchitecture (< 1.31); for females, normal (> 1.35), partially reduced bone microarchitecture (1.27–1.35), and reduced bone microarchitecture (< 1.27) (9); The meta-analysis reference range for TBS (L1–4) was categorized as normal (TBS ≥ 1.35), partially reduced bone microarchitecture (1.21–1.34), reduced bone microarchitecture (≤ 1.20) (8).

### Statistical analysis

Data are expressed as means ± standard deviation (SD). The fracture incidence was calculated by dividing the number of patients with vertebral fractures by the total number of participants in each group. The normality of continuous variables was assessed using the Shapiro-Wilk test. TBS followed a normal distribution, whereas LS-BMD and FN-BMD did not. Therefore, Student’s *t*-test was used to compare TBS, while the Wilcoxon rank-sum test was employed to compare LS-BMD and FN-BMD among the groups. Pearson correlation coefficient was used to analyze the correlation between variables. Chi-square or Fisher’s exact test was applied to compare proportions between groups. To evaluate differences in DXA and TBS diagnostic results across groups, the Kruskal-Wallis test or ANOVA was used. Additionally, ROC curve analysis was performed to calculate the area under the curve (AUC) to assess the predictive performance of the fracture model. Statistical analyses were performed using SPSS software (version 26.0; SPSS Inc., Chicago, United States), and a *P*-value < 0.05 was regarded as significant.

## Results

### Subject characteristics

**FIGURE 2:**
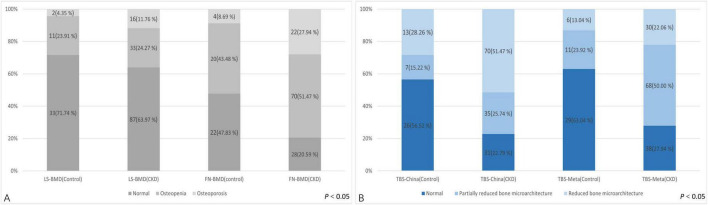
Difference in the TBS between the control group and CKD patients according to the diagnosis of the lumbar spine or femoral neck.

**FIGURE 3:**
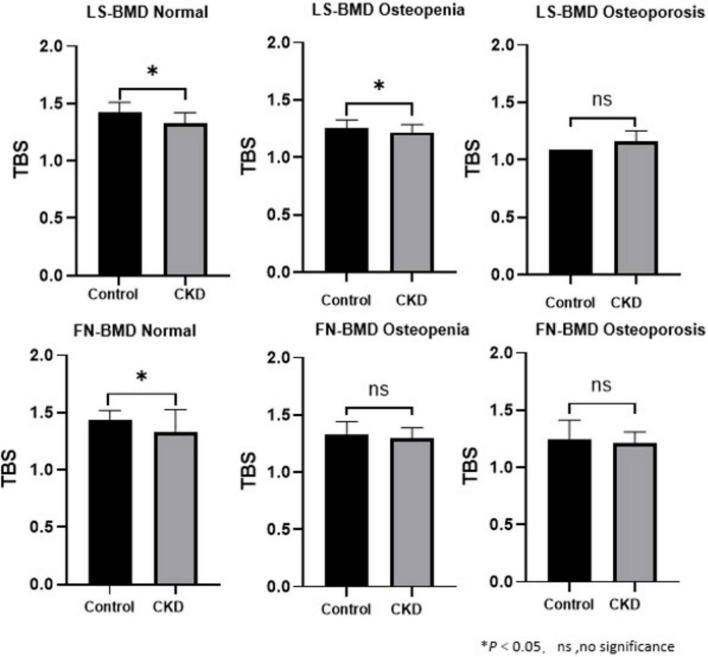
Analysis of Pearson correlation coefficients for BMD, TBS in CKD patients.

The baseline demographic, clinical, and laboratory characteristics of the control and CKD groups, with matching in age, gender, and body mass index (BMI) are summarized (Table 1). The average age of participants in the control group was 64.96 ± 7.76 years, with 27 females (58.70%), while the average age in the CKD group was 64.42 ± 10.90 years, with 66 females (48.53%). In the CKD group, creatinine (Cr), blood urea nitrogen (Urea), cystatin C, alkaline phosphatase (ALP), iPTH, and phosphorus (P) levels were significantly higher than those in the control group (P < 0.01). Conversely, the CKD group showed significantly lower eGFR, calcium (Ca), and plasma 25-(OH)-vitamin D levels compared to the control group (*P* < 0.01). In the CKD patient group, 74 patients had diabetes, 43 patients had fractures, 14 patients were smokers, and 8 patients were drinkers. In CKD patients, LS-BMD, FN-BMD, and TBS (L1–4) were significantly negatively correlated with BMI (*P* < 0.01); only TBS (L1–4) was negatively correlated with age (*P* < 0.01); no significant correlation was observed between LS-BMD, FN-BMD, TBS (L1–4), and renal function or bone turnover markers (Table 2). Differences in TBS were observed between groups classified based on LS-BMD or FN-BMD, irrespective of whether they were in the control group or the CKD group. TBS decreased as the severity of the bone density classification increased (*P* < 0.01) (Table 3; Figure 2). In the LS-BMD classification, significant differences in TBS were observed between the normal and osteopenia groups in both the control and CKD groups, with TBS being consistently lower in the CKD group. Similarly, in the FN-BMD classification, the normal groups also exhibited significant differences in TBS, with lower values observed in the CKD group (Figure 3).

**TABLE 1:** Charsubjects and patients with CKD.

Characteristics	Control	CKD	*P-*value
No. of patients	*n* = 46	*n* = 136	–
Age (year)	64.96 ± 7.76	64.42 ± 10.90	0.758
Sex (Female)	46(27)	136(66)	0.233(χ^2^)
Height (cm)	162.09 ± 7.54	159.85 ± 7.79	0.982
Weight (kg)	64.83 ± 12.32	58.10 ± 11.45	0.133
BMI (kg/m^2^)	23.53 ± 3.45	22.65 ± 3.66	0.156
Fracture	–	43	–
Diabetes mellitus	–	74	–
Smoking	–	14	–
Alcohol ≥ 3 units/day	–	8	–
**Laboratory values**			
eGFR (mL/min/1.73 m^2^)	–	16.29 ± 19.44	–
Creatinine (μmol/L)	74.05 ± 18.18	549.64 ± 328.57	<0.01
Urea (mmol/L)	4.69 ± 1.04	17.78 ± 8.44	<0.01
Cystatin C (mg/L)	0.78 ± 0.15	4.50 ± 2.01	<0.01
ALP (U/L)	7.12 ± 1.85	26.58 ± 49.84	<0.01
25-(OH) vitamin D (ng/mL)	20.64 ± 5.47	18.01 ± 8.03	<0.05
iPTH (pg/mL)	33.82 ± 16.58	333.78 ± 529.19	<0.01
Ca (mmol/L)	2.34 ± 0.13	2.14 ± 0.29	<0.01
P (mmol/L)	1.21 ± 0.16	1.62 ± 0.54	<0.01
**Diagnostic criteria**			
Overall BMD (normal:osteopenia:osteoporosis)	(22:19:5)	(27:68:41)	<0.001(χ^2^)
LS-BMD (normal:osteopenia:osteoporosis)	(29:11:2)	(87:33:16)	0.421(χ^2^)
FN-BMD (normal:osteopenia:osteoporosis)	(22:20:4)	(28:70:38)	<0.001(χ^2^)
META’s TBS reference (normal:Partially reduced bone microarchitecture:Reduced bone microarchitecture)	(29:11:2)	(38:68:30)	<0.001(χ^2^)
Chinese TBS reference (normal:Partially reduced bone microarchitecture:Reduced bone microarchitecture)	(26:7:13)	(31:35:70)	<0.001(χ^2^)
**BMD-related values**			
LS-BMD (mg/cm^2^)	1.04 ± 0.20	1.04 ± 0.22	0.891
FN-BMD (mg/cm^2^)	0.89 ± 0.15	0.73 ± 0.14	<0.01
TBS	1.37 ± 0.12	1.28 ± 0.11	<0.01

BMI, body mass index; eGFR, estimated glomerular filtration rate estimate by the original modification of diet in renal disease equation; ALP, alkaline phosphatase; Ca, Calcium; P, phosphate; iPTH, intact parathyroid hormone; LS-BMD, Lumbar vertebrae bone mineral density; FN-BMD, Femoral neck bone mineral density; TBS, Trabecular bone score. Variables are expressed as mean ± the standard deviation. The χ^2^ test was used to compare the number of the patients in groups.

**TABLE 2:** Correlation between clinical findings and bone parameters.

Variable	LS-BMD		FN-BMD		TBS	
	β	*P*-value	β	*P-*value	β	*P*-value
Age (year)	–0.029	0.734	–0.135	0.118	–0.301	<0.001
BMI	0.397	<0.001	0.452	<0.001	0.203	0.018
eGFR (mL/min/1.73 m^2^)	0.114	0.185	0.119	0.169	0.061	0.482
Creatinine (umol/L)	–0.065	0.453	–0.034	0.695	0.019	0.824
Calcium (Ca)	0.015	0.861	–0.066	0.446	0.048	0.577
Phosphate (P)	–0.095	0.273	0.023	0.789	–0.012	0.893
25-OHvitamin D (ng/mL)	0.013	0.881	0.066	0.448	0.141	0.102
ALP (U/L)	–0.078	0.368	–0.052	0.547	0.028	0.748
iPTH (pg/mL)	–0.123	0.154	–0.116	0.180	0.006	0.944
Urea (mmol/L)	–0.011	0.900	–0.011	0.901	0.125	0.148
Cystatin C (mg/L)	–0.025	0.771	–0.150	0.081	–0.033	0.704
LS-BMD (mg/cm^2^)	–	–	0.665	<0.001	0.667	<0.001
FN-BMD (mg/cm^2^)	0.665	<0.001	–	–	0.556	<0.001
TBS	0.667	<0.001	0.556	<0.001	–	–

**TABLE 3:** Comparative baseline variables according to BMD status from LS-BMD and FN-BMD of the CKD subjects.

Characteristics	LS-BMD				FN-BMD			
	Normal	Osteopenia	Osteoporosis	*p* value	Normal	Osteopenia	Osteoporosis	*p-*value
No. of patients	87	33	16	–	28	70	38	–
Age (years)	64.09 ± 10.62	65.42 ± 12.16	64.13 ± 10.16	0.833	60.39 ± 11.07	65.21 ± 10.31	65.92 ± 11.40	0.085
Gender				0.86				0.02
Male	57	23	10		14	41	15	
Female	30	10	6		14	29	23	
BMI (kg/m^2^)	23.56 ± 3.77^b,c^	21.97 ± 2.75 ^a,c^	19.13 ± 1.93 ^a,b^	<0.001	22.46 ± 3.91 ^c^	24.44 ± 3.83 ^c^	21.84 ± 3.15 ^a,b^	<0.001
eGFR (mL/min/1.73 m^2^)	17.47 ± 19.60	14.69 ± 21.07	10.08 ± 11.95	0.094	13.89 ± 14.33	18.58 ± 23.21	16.20 ± 19.48	0.331
Creatinine (μmol/L)	536.05 ± 351.89	548.19 ± 284.98	626.54 ± 284.71	0.404	578.54 ± 336.19	555.02 ± 340.93	534.15 ± 322.76	0.571
Ca (mmol/L)	2.12 ± 0.27	2.13 ± 0.33	2.26 ± 0.30	0.352	2.16 ± 0.28	2.19 ± 0.25	2.11 ± 0.31	0.105
P (mmol/L)	1.61 ± 0.54	1.61 ± 0.49	1.72 ± 0.62	0.859	1.57 ± 0.60	1.72 ± 0.46	1.59 ± 0.55	0.541
25-(OH) vitamin D (ng/mL)	18.30 ± 8.21	18.07 ± 8.39	16.26 ± 6.37	0.617	19.41 ± 7.92	18.15 ± 6.06	17.31 ± 8.92	0.23
ALP (U/L)	17.95 ± 18.11	25.35 ± 48.10	51.08 ± 87.08	0.161	33.38 ± 61.40	26.77 ± 58.69	23.47 ± 38.65	0.067
iPTH (pg/mL)	324.21 ± 503.37	208.69 ± 260.46	643.77 ± 886.28	0.161	414.22 ± 659.29	361.60 ± 581.77	281.23 ± 424.44	0.061
Urea (mmol/L)	17.93 ± 9.39	18.27 ± 6.76	15.96 ± 5.81	0.609	19.84 ± 11.15	17.24 ± 7.79	17.13 ± 7.27	0.728
Cystatin C (mg/L)	4.34 ± 2.05	4.47 ± 1.99	5.46 ± 1.59	0.071	4.63 ± 1.96	4.63 ± 2.31 ^c^	4.38 ± 1.88 ^b^	0.008
LS-BMD (mg/cm^2^)	1.17 ± 0.17 ^b,c^	0.89 ± 0.05 ^a,c^	0.71 ± 0.05 ^a,b^	<0.001	1.25 ± 0.17 ^b,c^	1.10 ± 0.19 ^a,c^	0.93 ± 0.18 ^a,b^	<0.001
FN-BMD (mg/cm^2^)	0.80 ± 0.80 ^b,c^	0.64 ± 0.10 ^a^	0.57 ± 0.09 ^a^	<0.001	0.84 ± 0.82 ^b,c^	0.79 ± 0.19 ^a,c^	0.65 ± 0.12 ^a,b^	<0.001
TBS	1.32 ± 0.10 ^b,c^	1.21 ± 0.07 ^a^	1.16 ± 0.09 ^a^	<0.001	1.41 ± 0.04 ^c^	1.33 ± 0.03 ^c^	1.19 ± 0.07 ^a,b^	<0.001

BMI, body mass index; eGFR, estimated glomerular filtration rate estimate by the original modification of diet in renal disease equation; ALP, alkaline phosphatase; Ca, Calcium; P, phosphate; iPTH, intact parathyroid hormone; LS-BMD, Lumbar vertebrae bone mineral density; FN-BMD, Femoral neck bone mineral density; TBS, Trabecular bone score.Variables are expressed as mean ± the standard deviation. *p < 0.05, for comparison among three groups using Kruskal-Wallis test or ANCOVA test, ^a^*p* < 0.05, compared with the Normal group, ^b^*p* < 0.05, compared with the Osteopenia group, ^b^*p* < 0.05, compared with the Osteoporosis group.

### The diagnostic classification differences of BMD and TBS measurements in control group and CKD group

Regarding the diagnostic classification of BMD, there was no significant difference in LS-BMD between the control and CKD groups (1.04 ± 0.20 vs. 1.04 ± 0.22, *P* = 0.891). However, the FN-BMD in the CKD group was significantly lower than in the control group (0.73 ± 0.14 vs. 0.89 ± 0.15, *P* < 0.01), and the TBS (L1–4) was also lower in the CKD group compared to the control group (1.28 ± 0.11 vs. 1.37 ± 0.12, *P* < 0.01) (Table 1). No significant difference was found between the two groups in the proportion of individuals classified as normal, osteopenia, or osteoporosis based on LS-BMD T-scores (*P* = 0.421) (Table 3). However, the classification by Overall BMD T-scores and FN-BMD T-scores significantly differed from the control group, with a higher proportion of individuals classified as osteopenia or osteoporosis in the CKD group (*P* < 0.001).

**FIGURE 4:**
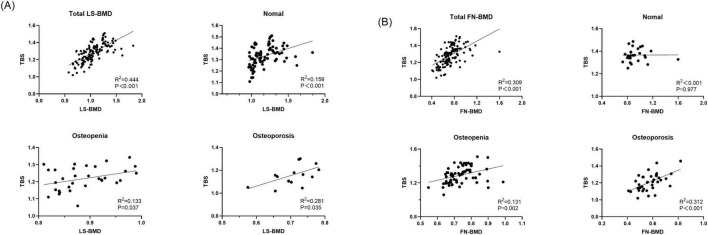
Discrepant diagnostic results of BMD and TBS for control group and CKD group. **(A)** Discrepant diagnostic in BMD. **(B)** Discrepant diagnostic in BMD.

Regarding the diagnostic classification of TBS, when classified using TBS (China/META reference ranges) as normal, partially reduced bone microarchitecture, or reduced bone microarchitecture, the proportion of reduced bone microarchitecture was significantly higher in the CKD group compared to the control group (*P* < 0.001) (Figure 4). In the control group, using the TBS (China reference range) standard classification, 26 subjects (56.52%) were evaluated as normal, 7 (15.22%) as partially reduced bone microarchitecture, and 13 (28.26%) as reduced bone microarchitecture; based on the TBS (META reference range) standard classification, 29 subjects (63.04%) were assessed as normal, 11 (23.91%) as partially reduced bone microarchitecture, and 2 (4.35%) as reduced bone microarchitecture (Table 4). The inconsistency in diagnoses between TBS (China reference range) and TBS (META reference range) was mainly observed when TBS (META reference range) was diagnosed as normal or partially reduced bone microarchitecture, 10 patients were diagnosed with partially reduced bone microarchitecture or reduced bone microarchitecture by TBS (China reference range) (*P* < 0.01) (Table 5).

**TABLE 4:** Difference of diagnosis in each group with discordant results of BMD and TBS.

	TBS Scores (Chinese TBS reference)				*P*-value	TBS Scores (Meta’s TBS reference)				*P*-value
Group	Normal	Partially reduced bone micro architecture	Reduced bone micro architecture	Total		Normal	Partially reduced bone micro architecture	Reduced bone micro architecture	Total	
**Control group**										
Overall BMD					<0.01					<0.01
Normal	17	3	2	22		18	4	0	22	
Osteopenia	8	4	7	19		10	6	3	19	
Osteoporosis	1	0	4	5		1	1	3	5	
Total	26	7	13	46		29	11	6	46	
LS BMD					<0.01					<0.01
Normal	26	4	3	33		28	4	1	33	
Osteopenia	0	3	8	11		1	7	3	11	
Osteoporosis	0	0	2	2		0	0	2	2	
Total	26	7	13	46		29	11	6	46	
FN BMD										
Normal	17	3	2	22	0.014	18	4	0	22	<0.01
Osteopenia	8	4	8	20		10	6	4	20	
Osteoporosis	1	0	3	4		1	1	2	4	
Total	26	7	13	46		29	11	6	46	
**CKD group**										
Overall BMD					<0.01					<0.01
Normal	15	8	4	27		16	10	1	27	
Osteopenia	14	20	34	68		19	39	10	68	
Osteoporosis	2	7	32	41		3	19	19	41	
Total	31	35	70	136		38	68	30	136	
LS BMD					<0.01					<0.01
Normal	31	28	28	87		38	42	7	87	
Osteopenia	0	4	29	33		0	21	12	33	
Osteoporosis	0	3	13	16		0	5	11	16	
Total	31	35	70	136		38	68	30	136	
FN BMD					<0.01					<0.01
Normal	15	9	4	28		16	11	1	28	
Osteopenia	14	20	36	70		19	39	12	70	
Osteoporosis	2	6	30	38		3	18	17	38	
Total	31	35	70	136		38	68	30	136	
**Fracture group**										
Overall BMD					<0.01					<0.05
Normal	5	3	2	10		5	5	0	10	
Osteopenia	4	3	8	15		4	8	3	15	
Osteoporosis	0	2	16	18		1	8	9	18	
Total	9	8	26	43		10	21	12	43	
LS BMD					<0.01					<0.01
Normal	9	7	9	25		10	13	2	25	
Osteopenia	0	0	10	10		0	7	3	10	
Osteoporosis	0	1	7	8		0	1	7	8	
Total	9	8	26	43		10	21	12	43	
FN BMD					<0.01					<0.05
Normal	5	3	2	10		5	5	0	10	
Osteopenia	4	4	9	17		4	9	4	17	
Osteoporosis	0	1	15	16		1	7	8	16	
Total	9	8	26	43		10	21	12	43	

The χ^2^ test or Fisher test was used to compare the number of the patients grouped by TBS (Chinese TBS reference) and TBS (Meta’s TBS reference) in BMD (Normal/Osteopenia) and TBS (Partially reduced bone microarchitecture/Reduced bone microarchitecture) status.

**TABLE 5:** Difference of diagnosis in control group and CKD group with discordant results of Chinese TBS reference and Meta’s TBS reference.

Control group	TBS (Chinese TBS reference)				*P-*value
TBS (Meta’s TBS reference)	Normal	Partially reduced bone micro architecture	Reduced bone micro architecture	Total	<0.01
Normal	26	3	0	29	
Partially reduced bone microarchitecture	0	4	7	11	
Reduced bone microarchitecture	0	0	6	6	
Total	26	7	13	46	
**CKD group**					
TBS (META’s TBS reference)					<0.01
Normal	30	8	0	38	
Partially reduced bone microarchitecture	0	27	41	68	
Reduced bone microarchitecture	1	0	29	30	
Total	31	35	80	136	
**Fracture group**					
TBS (META’s TBS reference)					<0.01
Normal	9	1	0	10	
Partially reduced bone microarchitecture	0	7	14	21	
Reduced bone microarchitecture	0	0	12	12	
Total	9	8	26	43	

The χ^2^ test or Fisher test was used to compare the number of diagnostic classification discrepancies between TBS (Chinese TBS reference) and TBS (Meta’s TBS reference).

In the CKD group, using the TBS (China reference range) classification, 31 subjects (22.79%) were assessed as normal, 35 (25.74%) as partially reduced bone microarchitecture, and 70 (51.47%) as reduced bone microarchitecture; according to the TBS (META reference range) classification, 38 subjects (27.94%) were evaluated as normal, 68 (50.00%) as partially reduced bone microarchitecture, and 30 (22.06%) as reduced bone microarchitecture (Table 4). Whether the Overall BMD T-score, LS-BMD T-score, or FN-BMD T-score is diagnosed as normal or osteopenia, some patients were diagnosed with partially reduced bone microarchitecture or reduced bone microarchitecture based on TBS (China/META reference range) (*P* < 0.05). The inconsistency between TBS (China reference range) and TBS (META reference range) diagnoses was mainly observed when TBS (META reference range) diagnosed as normal or partially reduced bone microarchitecture, 49 patients were diagnosed with partially reduced bone microarchitecture damage or reduced bone microarchitecture by TBS (China reference range) (*P* < 0.01) (Table 5).

### The diagnostic classification differences of BMD and TBS measurements in fracture participants of the CKD group

In CKD fracture patients, regardless of whether Overall BMD T-scores, LS-BMD T-scores, or FN-BMD T-scores were classified as normal or osteopenia, some patients were diagnosed with partial or complete trabecular bone degradation based on either TBS (China reference range) or TBS (META reference range). When fracture patients were diagnosed as normal or osteopenia based on Overall BMD T-scores, LS-BMD T-scores, or FN-BMD T-scores, a higher number of fracture patients were diagnosed with partial or complete trabecular bone degradation according to TBS (China reference range) compared to TBS (META reference range), Percentages: [13 (30.23%) vs. 8 (18.60%), 26 (60.47%) vs. 18 (41.86%), 14 (32.56%) vs. 9 (20.93%)] (*P* < 0.01) (Table 4). Among the 43 CKD fracture patients, 15 patients classified as normal or partially reduced bone microarchitecture according to TBS (META reference range) were classified as having partially reduced bone microarchitecture or reduced bone microarchitecture according to TBS (China reference range) (*P* < 0.01) (Table 5).

### Comparison of AUC for fracture prediction in the CKD group based on bone parameters

In the Overall BMD (Normal/Osteopenia) and Overall BMD (Osteoporosis) groups, the combination model of TBS, FN-BMD, and age significantly enhanced the ability to distinguish fractures. In the LS-BMD (Normal/Osteopenia) group, the TBS, FN-BMD, and age model showed fracture discrimination capability comparable to the TBS, LS-BMD, FN-BMD, and age model (*P* < 0.05). In the LS-BMD (Osteoporosis) group, the combination of TBS, LS-BMD, FN-BMD, and age significantly enhanced fracture discrimination (*P* < 0.05). In the FN-BMD (Normal/Osteopenia) group, none of the models showed significant differences in fracture discrimination. In the FN-BMD (Osteoporosis) group, models combining FN-BMD, LS-BMD, and age; TBS, FN-BMD, and age; and TBS, LS-BMD, FN-BMD, and age effectively distinguished fractures, with the TBS, LS-BMD, FN-BMD, and age model having the highest AUC (*P* < 0.05). In the Overall BMD (Normal/Osteopenia/Osteoporosis) group, the TBS, FN-BMD, and age model demonstrated similar fracture discrimination capability to the TBS, LS-BMD, FN-BMD, and age model, effectively enhancing fracture discrimination (*P* < 0.05) (Table 6).

**TABLE 6:** Comparison of AUC for prediction of vertebral fracture according to WHO classification.

T-score	AUC	SE	95% CI	*P*-value (vs. Model 1)
**Overall BMD (Normal/Osteopenia)**				
Model 1	0.62	0.06	0.49–0.74	–
Model 2	0.62	0.06	0.5–0.74	0.08
Model 3	0.63	0.06	0.51–0.75	0.05
Model 4	0.62	0.06	0.50–0.74	0.07
**Overall BMD (Osteoporosis)**				
Model 1	0.64	0.09	0.46–0.82	–
Model 2	0.64	0.09	0.46–0.82	0.13
Model 3	0.68	0.09	0.50–0.85	0.05
Model 4	0.67	0.09	0.50–0.84	0.06
**LS-BMD (Normal/Osteopenia)**				
Model 1	0.60	0.06	0.49–0.71	–
Model 2	0.60	0.06	0.49–0.71	0.09
Model 3	0.62	0.06	0.51–0.73	0.04
Model 4	0.62	0.06	0.51–0.73	0.03
**LS-BMD (Osteoporosis)**				
Model 1	0.64	0.15	0.34–0.94	–
Model 2	0.67	0.14	0.39–0.96	0.25
Model 3	0.73	0.13	0.47–1.00	0.12
Model 4	0.83	0.12	0.59–1.00	0.03
**FN-BMD (Normal/Osteopenia)**				
Model 1	0.60	0.06	0.47–0.72	–
Model 2	0.60	0.06	0.47–0.72	0.14
Model 3	0.60	0.06	0.48–0.73	0.12
Model 4	0.60	0.06	0.48–0.73	0.12
**FN-BMD (Osteoporosis)**				
Model 1	0.69	0.09	0.51–0.87	–
Model 2	0.71	0.09	0.54–0.88	0.03
Model 3	0.71	0.09	0.53–0.88	0.03
Model 4	0.73	0.08	0.57–0.90	0.02
**Overall BMD (Normal/Osteopenia/Osteoporosis)**				
Model 1	0.59	0.05	0.49–0.70	–
Model 2	0.59	0.05	0.49–0.69	0.10
Model 3	0.61	0.05	0.51–0.71	0.04
Model 4	0.61	0.05	0.51–0.71	0.04
**AUC, area under curve; CI, confidence interval; TBS, trabecular bone score; LS,lumbar vertebra; FN, femur neck.**				
Model 1:FN-BMD + AGE				
Model 2:FN-BMD + AGE + LS-BMD				
Model 3:FN-BMD + AGE + TBS				
Model 4:FN-BMD + AGE + LS-BMD + TBS				

## Discussion

In CKD patients, chronic kidney damage combined with factors like abnormal protein metabolism, insufficient vitamin D production, and secondary hyperparathyroidism causes severe mineral imbalances, leading to bone metabolism disorders. The resulting low bone turnover state is typically accompanied by higher bone quality than that of age- and sex-matched populations (13). The results of studies evaluating bone parameters (such as BMD, TBS, etc.) in CKD patients are inconsistent, and no medical consensus has been reached (14, 15), especially in dialysis-naïve CKD patients with normal BMD but reduced TBS, where further research is lacking. Additionally, the reference ranges for TBS in bone parameters vary by region, and most studies focus primarily on dialysis patients, with limited attention given to pre-dialysis CKD patients. Hence, the aim of this study is to analyze the relationship between bone parameters and pre-dialysis CKD patients in China, and compare the differences in bone quality between CKD patients classified by the Chinese TBS reference range and those classified by the META TBS reference range.

We confirmed that there were significant differences between CKD patients and the control group in the proportions of participants classified based on Overall BMD, FN-BMD, and TBS; the FN-BMD and TBS values also differed significantly between the two groups. However, LS-BMD showed no significant differences, either in the proportions classified by T-score or in the LS-BMD values. We hypothesize that the inconsistency in the results for CKD patients reflects the limitations of using only DXA-derived BMD measurements. The reason for this may be that LS-BMD is influenced by vertebral body size and pathological conditions, such as osteoarthritis, vertebral osteophytes, and abdominal aortic calcification, whereas FN-BMD and TBS are not affected by these factors (16, 17). In our study, TBS did not show a significant correlation with bone turnover parameters, which is similar to some previous findings (18). This suggests that bone microstructure deterioration and increased fracture risk may depend on factors other than high bone turnover. However, some studies have shown a link between TBS and bone turnover markers^13^, warranting further research. In our study, BMI was correlated with bone parameters (LS-BMD *r* = 0.40, *P* < 0.001; FN-BMD *r* = 0.45, *P* < 0.001; TBS *r* = 0.20, *P* = 0.02). As a result, the increase in adipose tissue around the region of interest may reduce the signal-to-noise ratio, leading to lower bone parameter values. Notably, the BMI of our CKD patients fell within the recommended working range for TBS (15–37 kg/m^2^).

In this study, TBS was significantly correlated with both LS-BMD and FN-BMD. As BMD decreased, the correlation between TBS and LS-BMD, FN-BMD strengthened. In our study, when patients were classified based on TBS scores into osteopenia, osteoporosis, and normal BMD categories, significant differences were observed. Patients with lower Overall BMD, LS-BMD, and FN-BMD had a higher proportion of TBS falling into partially reduced bone microarchitecture or reduced bone microarchitecture categories. In our study, TBS changed the diagnosis of Overall BMD, LS-BMD, and FN-BMD to a worse classification in the majority of CKD patients. Even in patients with normal BMD, lower TBS in CKD patients was clinically significant. When BMD was classified as normal or osteopenia, some patients had already sustained fractures, and their TBS was diagnosed as partially reduced bone microarchitecture or reduced bone microarchitecture. Our study demonstrates that TBS levels have predictive value for fractures not only in individuals diagnosed with osteoporosis by BMD but also in the overall population diagnosed with normal or osteopenia LS-BMD. While it is commonly believed that patients with normal BMD have fewer fractures, clinical observations show that many fracture patients actually present with normal BMD. Specifically, in this study, TBS was more predictive of fractures than BMD. This implies that CKD patients with normal bone density but reduced TBS may be more susceptible to fractures. In our study, we found that the combination of the TBS model significantly improved the ability to identify fractures in CKD patients. This may be attributed to the fact that our study population consisted of postmenopausal women and men over the age of 50, some of whom may have experienced degenerative changes in the spine. TBS, however, is not affected by these spinal degenerative changes. In contrast, BMD fails to capture the complex microstructural abnormalities associated with CKD-MBD, which can increase bone fragility and often lead to an underestimation of fracture risk. Additionally, TBS reflects the trabecular microstructure in CKD patients’ bone, and studies have shown that trabecular bone has a larger surface area and greater mineral buffering capacity compared to cortical bone (15, 19). Therefore, the model incorporating TBS significantly improves fracture discrimination in CKD patients.

The occurrence of fractures is the result of a multifactorial process, where BMD is an important factor in evaluating bone strength and fracture risk (including vertebral fractures), but bone quality is also crucial (20). Even in populations with osteopenia or normal BMD, the high fracture incidence may highlight the importance of bone quality (21). Bone quality is a multifaceted composite metric that involves trabecular microstructure, cortical bone properties, mineralization levels, metabolic state, bone marrow environment, nutritional status, and more. Alterations in these factors may independently or interactively affect bone mechanical properties and fracture resistance, ultimately leading to an increased fracture risk (22). Recent studies have also indicated that TBS reflects the trabecular microstructure of bone in CKD patients (23), and TBS measurement has been shown to be highly valuable in detecting fractures in patients with secondary osteoporosis (24). In CKD kidney transplantation studies, it was also found that when BMD is normal, TBS can reflect the fracture risk of patients (25). This phenomenon may be attributed to the complexity of CKD, where alterations in bone metabolism reflect multiple physiological processes (such as osteomalacia, hypoactive bone disease, and mixed bone disease), resulting in normal bone strength despite reduced bone quality (26), thereby increasing the fracture risk in CKD patients.

Currently, there is no standard unified reference range for TBS. The most commonly used reference range is derived from a meta-analysis (8), while some countries or regions, such as the United States, Mexico, Australia, Japan, and China, have developed their own local reference ranges to account for confounding factors like race and region (9, 27–29). Therefore, this study is the first to use the TBS China reference range to assess CKD patients in China. In our study, the number of patients classified with partial or complete bone loss based on the Chinese reference range was higher than those classified using the international reference range. Additionally, when BMD was diagnosed as normal or osteopenia, the number of fractures classified as having partially reduced bone microarchitecture or reduced bone microarchitecture using the Chinese reference range was higher than those classified with the international reference range. This indicates that the TBS classification based on the Chinese reference range has better predictive ability for fractures where diagnoses are inconsistent. It also underscores the importance of establishing regional TBS reference ranges for clinicians to evaluate the bone status of CKD patients.

This study has some limitations. First, it is a single-center retrospective study with a small sample size, and the strength of evidence is not as strong as that of a prospective study. To improve the broader applicability of our results, future research should focus on multi-center and prospective studies involving larger cohorts. Second, due to the limitation in sample size, we were unable to conduct further stratified analysis of pre-dialysis CKD patients with varying degrees of renal dysfunction to explore the bone status under different renal function conditions. Third, our study did not classify fractures or investigate their influencing factors, although some studies have shown that TBS is related to non-vertebral fractures (13). This is mainly because the number of hip fractures among the patients we included was only 3 cases, and future research needs to further expand the sample size.

In conclusion, TBS is lower in CKD patients compared to the control group, and TBS is positively correlated with BMD. For situations where there is a discrepancy with BMD diagnosis, TBS has a higher predictive value for fractures in CKD patients. Furthermore, the TBS classification based on the Chinese reference range is more strongly associated with fracture rates in CKD patients. These findings support the use of TBS as a tool for identifying fracture risk in CKD patients. However, further studies with larger sample sizes are needed to validate the conclusions of this study.

## Data Availability

The raw data supporting the conclusions of this article will be made available by the authors, without undue reservation.
